# Precise local control of liquid crystal pretilt on polymer layers by focused ion beam nanopatterning

**DOI:** 10.3762/bjnano.10.164

**Published:** 2019-08-12

**Authors:** Maxim V Gorkunov, Irina V Kasyanova, Vladimir V Artemov, Alena V Mamonova, Serguei P Palto

**Affiliations:** 1 Shubnikov Institute of Crystallography, FSRC “Crystallography and Photonics”, Russian Academy of Sciences, 119333 Moscow, Russia; 2 National Research Nuclear University MEPhI (Moscow Engineering Physics Institute), 115409 Moscow, Russia

**Keywords:** focused ion beam nanopatterning, nematic liquid crystal, optical retardation, pretilt control, surface anchoring

## Abstract

**Background:** The alignment of liquid crystals by surfaces is crucial for applications. It determines the director configuration in the bulk, its stability against defects and electro-optical switching scenarios. The conventional planar alignment of rubbed polymer layers can be locally flipped to vertical by irradiation with a focused ion beam on a scale of tens of nanometers.

**Results:** We propose a digital method to precisely steer the liquid crystal director tilt at polymer surfaces by combining micrometer-size areas treated with focused ion beam and pristine areas. The liquid crystal tends to average the competing vertical and planar alignment actions and is stabilized with an intermediate pretilt angle determined by the local pattern duty factor. In particular, we create micrometer-sized periodic stripe patterns with this factor gradually varying from 0 to 1. Our optical studies confirm a predictable alignment of a nematic liquid crystal with the pretilt angle continuously changing from 0° to 90°. A one-constant model neglecting the difference between the elastic moduli reproduces the results quantitatively correctly.

**Conclusion:** The possibility of nanofabrication of polymer substrates supporting an arbitrary (from planar to vertical) spatially inhomogeneous liquid crystal alignment opens up prospects of “imprinting” electrically tunable versatile metasurfaces constituting lenses, prisms and q-plates.

## Introduction

Liquid crystal (LC) interactions with adjacent interfaces play a key role in LC-based optical devices as they are responsible for stabilizing mono-domain LC configurations and ensuring their reliable electro-optical switching [1]. The reason is of fundamental nature: Anisotropic LC phases arise due to collective intermolecular interactions in the LC bulk that allow for different LC orientations in the laboratory frame. Without external alignment, LC samples are randomly multi-domain and perform unpredictably. Surfaces remove the orientation degeneracy and establish a desired overall LC director distribution. Although particular microscopic mechanisms of the LC–surface interactions (so-called LC anchoring) can be of very different nature, for the modelling of macroscopic LC systems phenomenological approaches are usually sufficient [2]. One implies, for instance, a certain fixed director orientation at the surface (rigid boundary), or introduces a specific surface contribution to the LC free energy depending on the director orientation at the surface (soft boundary) [3]. In such terms, all physical and chemical properties of the surface are accounted for by a few phenomenological parameters, while causal relations between the surface preparation routine and its LC-anchoring action remain predominantly empirical. Accordingly, reliable practical recipes of establishing particular LC alignments are highly valuable.

Thin polymer layers induce either vertical alignment of nematic LCs, with the director oriented orthogonally to the interface plane, or planar alignment, with the director aligned in parallel to this plane. It was empirically established half a century ago that the type of alignment is related to the difference of surface energy between the LC and the polymer [4–5]. The underlying microscopic mechanisms, however, are yet not fully clarified and valuable related facts are being continuously accumulated [6].

A planar aligning surface reduces the director orientation degeneracy but does not eliminate it. Introducing an unambiguous in-plane direction requires additional steps, such as mechanical unidirectional rubbing [7], irradiation with polarized light [8], or bombardment with ions at glancing angles [9]. Typically, the result is rigid anchoring of the LC director along a direction that is not truly in-plane, but may point at a certain small angle away from it depending on the technological parameters of the surface preparation routine [10]. Intermediate (neither planar nor vertical) LC surface alignment is known to have certain advantages, as it, for example, helps avoiding topological defects [11] and accelerates switching of vertically aligned LC cells [12]. It is quantitatively characterized by the pretilt angle between the director and the surface plane. Surfaces stabilizing pretilted alignments remain rare and require more complex fabrication techniques such as patterning with nanogrooves [13–15] and nanoslits [16], ion-beam irradiation of specific inorganic [17] and polymer [18] substrates, subjecting of photo-controlled aligning polymers to near-threshold doses of ultraviolet radiation [19], formation of surface microdomains from segregating mixtures of vertically and planar aligning polymers [20–21], or stacking of nanolayers of such polymers [22]. The techniques yield homogeneous pretilt angles sufficient for conventional flat-display applications but are very inconvenient for establishing spatially inhomogeneous states. Being also essentially analog, the techniques rely on partial modification of the alignment material, and yield uncertain values of the pretilt angle indirectly affected by multiple external factors.

Numerous emerging non-display LC applications benefit from controllable inhomogeneous LC director distributions. LC-based diffraction gratings [23–26], lenses [27–28], q-plates [29], and holographic images [30] all require alternating surface anchoring conditions to stabilize complex director patterns in the LC bulk. Presently, those are realized by photoalignment [8] of organic layers that, upon irradiation with appropriate polarized optical interference patterns, induce planar LC alignment with the director rotating within the substrate plane.

Recently, we have reported on LC metasurfaces formed due to local transformation of polymer–LC anchoring from planar to vertical by focused ion beam (FIB) [31]. While the particular microscopic mechanism of this transformation is yet to be revealed, it is hard to underestimate the emerging application prospects. Modern FIB devices of deeply submicrometer resolution are digitally controlled with preprogrammed raster templates and can straightforwardly “imprint” arbitrary high-definition LC aligning patterns.

Here we demonstrate a particular practical benefit of this technique, i.e., the possibility to establish LC states with precisely predictable intermediate values of the pretilt angle. The latter arise as a result of averaging of two binary LC director states, planar and vertical, mixed in a desired proportion. Digital FIB allows us to accurately control this proportion, to avoid uncertainties related to intermediate treatment doses and to accurately steer the pretilt angle variation across the aligning surface.

## Results and Discussion

### Sample fabrication

We use commercial display-quality glass substrates covered with ca. 150 nm thin transparent ITO electrodes. Polyimide (PI) is deposited onto the substrates by a conventional routine combining a precursor spin-coating and annealing at 190 °C for 1 h, which produces a mechanically robust PI layer of 10–20 nm thickness. Next, the PI layer is unidirectionally rubbed with a cotton cloth. The empirically chosen rubbing regime is intensive enough to establish a uniform planar LC aligning action upon the whole substrate, but, at the same time, avoids substantial damage or partial removal of the PI layer.

The conversion of LC anchoring conditions from planar to vertical upon certain surface areas is performed using a FEI Scios dual-beam electron microscope operating with a FIB of Ga^+^ ions of a current of 0.1 nA accelerated by a voltage of 30 kV. The FIB patterning is controlled by digital templates, which prescribe the beam path within a raster consisting of up to 4096 × 3536 pixel^2^ as well as the time spent by the beam on each pixel (the so-called dwell time). According to our previous comparative study [31], a relatively small dose of Ga^+^ ions is sufficient for the required polymer transformation and we adopt here the same dwell time value of 150 μs.

**Figure 1:**
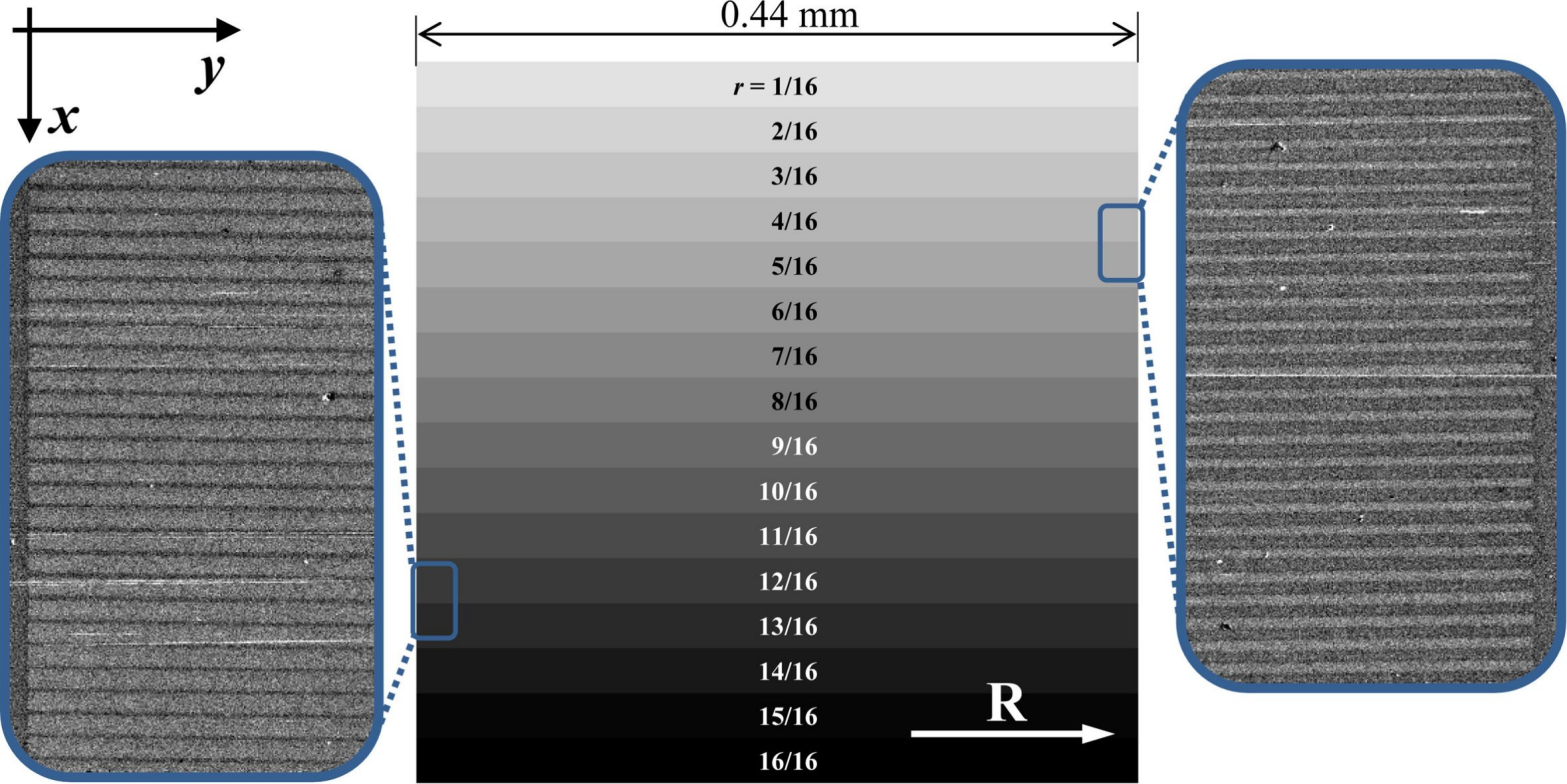
Schematic of a 2 μm periodic stripe pattern imprinted in the rubbed PI layer (rubbing direction shown by the white arrow) with the duty factor *r* gradually increasing downwards. The pattern comprises 220 periods, each corresponding to 16 pixels of the digital template. The discretized duty factor variation divides the pattern into 16 approximately equal areas (shown by the 16 shades of grey), corresponding to the indicated rational values of *r*. The insets show typical fragments of the scanning electron microscope images of the patterned PI.

To achieve intermediate LC pretilt, we imprint three periodic patterns of stripes parallel to the PI rubbing direction. The patterns cover square areas of 440–450 μm in size and consist of 440, 294 and 220 stripes imprinted with 1 μm, 1.5 μm and 2 μm periodicity, respectively. The chosen small periods allow us to minimize the effect of light diffraction by modulations of the LC and to establish LC configurations with effectively averaged contradictory aligning actions of rubbed and FIB-processed areas of PI. To combine those actions in different proportions, we imprint patterns with different duty factor *r*, which is the relative part of the PI area irradiated with FIB. Within all stripe patterns this factor is set to vary gradually from *r* = 0 on pristine PI to *r* = 1 on PI homogeneously irradiated with FIB. Because in the digital templates each period consists of 8, 12, and 16 pixels in 1 μm, 1.5 μm, and 2 μm periodic patterns, respectively, the duty factor values are naturally discretized as illustrated by Figure 1.

**Figure 2:**
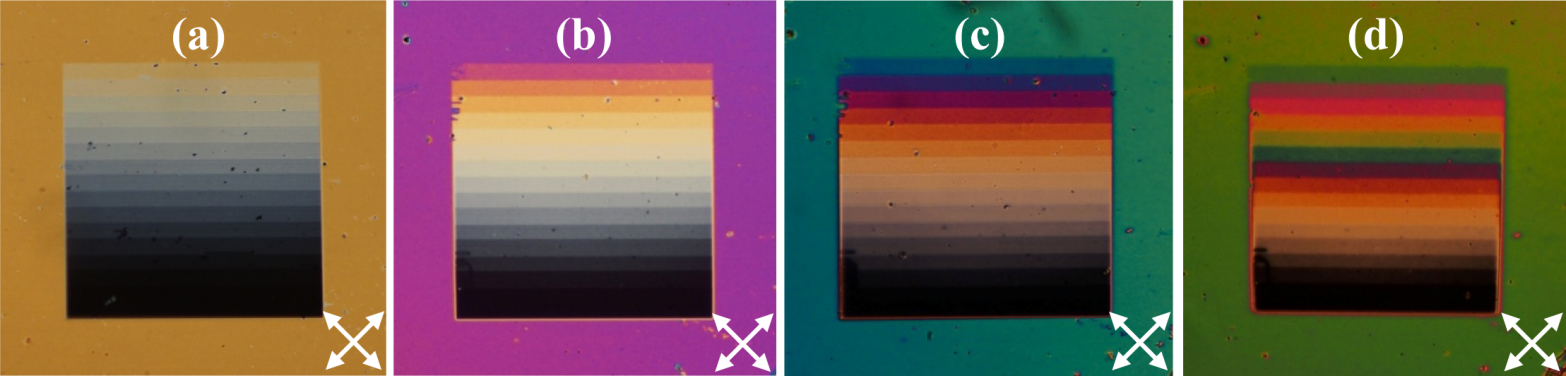
Exemplary PLM images in crossed polarisers (oriented as indicated by the white arrows) of the LC cells with the FIB-patterned area of 2 μm periodicity and the gradual duty factor variation for different LC layer thickness values: 3.8 μm (a), 5.0 μm (b), 6.5 μm (c) and 13 μm (d)

Next, LC cells are assembled by stacking the FIB-patterned substrate with another glass substrate, bearing an ITO layer and a vertically aligning layer of chromolane (spin-coated from 0.1 wt % ethanol solution and then dried at 95 °C). Note that in such a cell geometry, when the LC is vertically aligned at the top substrate, the difference between optical phase retardations of differently polarized normally incident light is fully determined by the LC orientation upon the patterned PI surface. Finally, the gap between the substrates fixed with Sekisui Micropearl spherical spacers is filled with a nematic liquid crystal material E7 (Merck). After being optically studied, the cells are repeatedly reassembled with spacers of different sizes allowing us to cover a broad range of the LC layer thickness *d* from 2.6 to 13 μm. For example, Figure 2b–d illustrates how the same patterned area aligns the LC inside cells of very different thickness.

### Optical studies

To reveal the LC alignment upon the patterned PI areas, we perform polarized light microscopy (PLM) studies using an Olympus CX31PF-5 polarized-light microscope. Typical images of a striped pattern inside LC cells of different thickness are shown in Figure 2. The images are obtained in crossed polarisers with the LC layer optical axis rotated by 45° with respect to the axes of the polarisers. The background colour arises due to the dispersion of phase retardation of the light propagating through the birefringent LC layer. We use the phase retardation value Γ_0_ measured at a wavelength of λ = 546 nm as a reference. In general, it is determined by the LC material birefringence Δ*n*, the LC layer thickness *d*, and the LC director distribution within the layer. For the particular LC cell with vertical alignment at one substrate and planar alignment at the other, the retardation is approximately Γ_0_ ≃ πΔ*nd*/λ, i.e., two times smaller than that for a uniform birefringent planar LC layer of the same thickness (see the modelling below).

On the PLM images of the LC cells in crossed polarisers one can clearly distinguish the patterned areas as sets of broad coloured stripes corresponding to the different rational duty factor values, see Figure 2. In all cases, independently of the pattern periodicity and cell thickness, the stripes with *r* = 1 remain black, as the LC optical axis is oriented uniformly vertically there. Other broad stripes exhibit distinct colours corresponding to the phase retardation values monotonically growing from zero to those of the background. The corresponding gradual colour variation is clearly seen in thinner cells depicted in Figure 2a–c. For the thicker cell shown in Figure 2d, one should take into account that the optical retardation of the background roughly equals 4π, and above the patterned areas the retardation grows from zero (above the bottom broad stripe) through the value of 2π (above broad stripe 12, counted from the bottom) up to a value close to 4π (above the top broad stripe 16). By adding a Berek compensator to the PLM setup we measure the phase retardation Γ_p_ at the same wavelength of 546 nm separately above each broad stripe.

**Figure 3:**
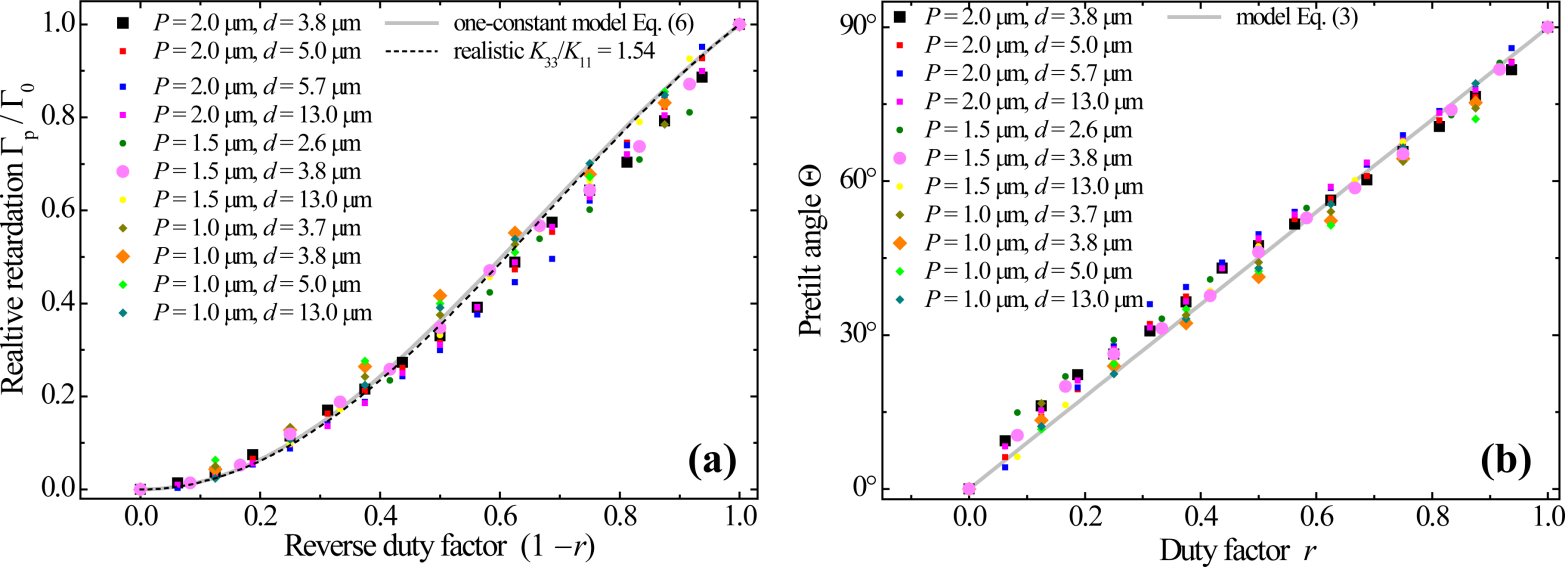
(a) The relative optical retardation (a) measured at a wavelength of 546 nm, and the pretilt angle (b) as functions of the duty factor for the nematic LC layers of different thickness *d* above the areas patterned with different periodicity *P* as indicated in the legends. The dependences given by Equation 1 and Equation 2 within the one-constant approximation are shown by the solid lines. The numerically calculated dependence for the elastic constants of E7 nematic is shown in (a) by the dashed line.

Remarkably, the resolved Γ_p_ values are practically independent of the pattern periodicity. Moreover, the relative retardation Γ_p_/Γ_0_, normalized by the background value Γ_0_ in the same cell, appears to be also independent of *d* and is solely controlled by the duty factor *r*. The data accumulated for the patterns of different periodicity in the LC cells of very different thickness are presented in Figure 3a. One can see a universal monotonic dependence of the relative birefringence on the reverse duty factor (1 − *r*) covering the whole range from 0 to 1, i.e., from the purely vertical LC alignment at *r* = 1 to the hybrid alignment as in the background at *r* = 0, when the LC director is vertical on one substrate and planar on the other.

### Model

**Figure 4:**
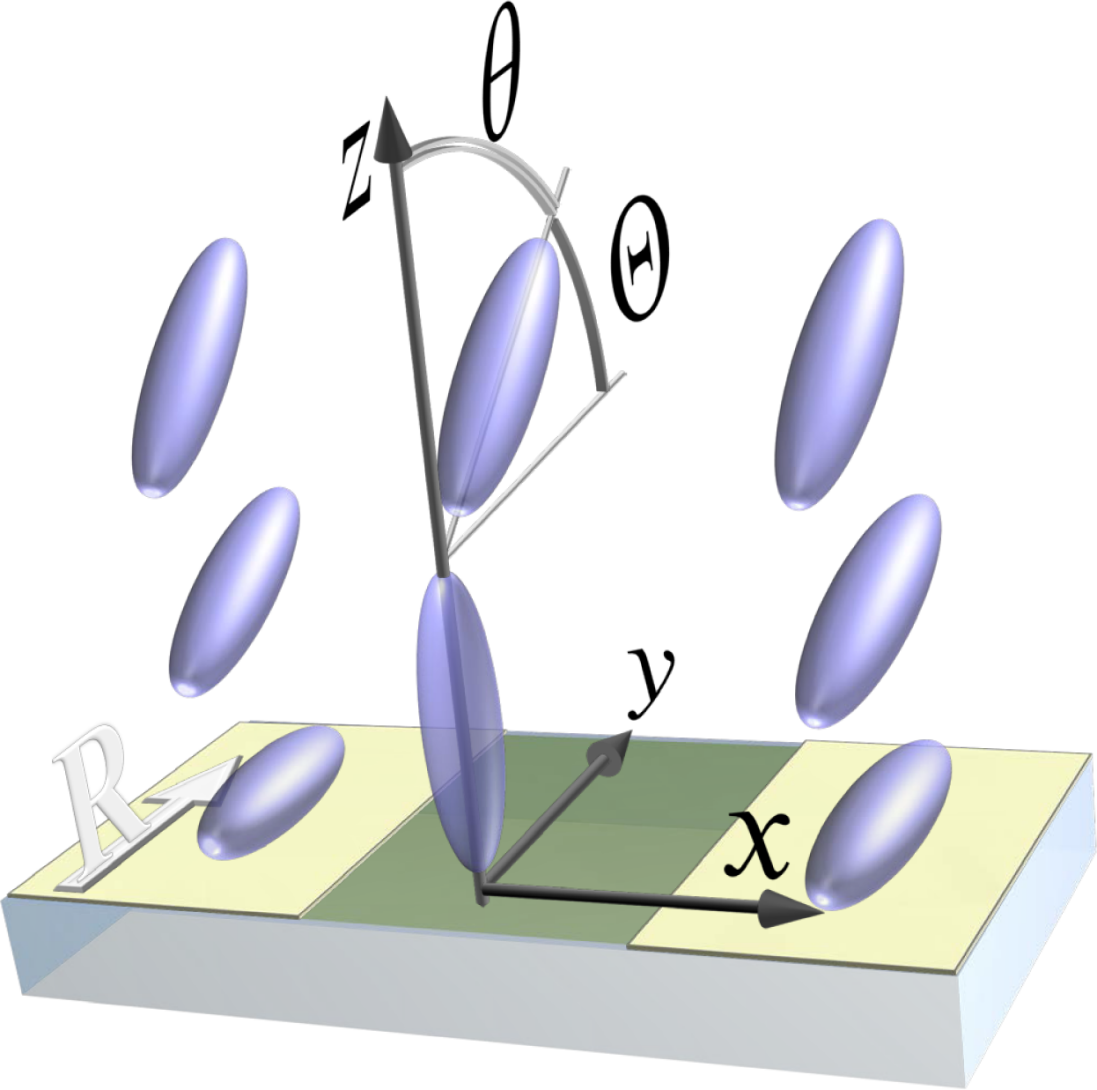
Schematic of the LC orientation on the striped pattern: The molecules are anchored in-plane along the rubbing direction (white “R” arrow) by pristine PI (light yellow stripes) and orthogonally to the substrate by PI irradiated with FIB (middle darker stripe). The competing aligning actions are averaged by the LC elasticity at some distance from the substrate and establish a homogeneous polar angle θ determining the pretilt angle Θ.

A sufficient theoretical explanation can be obtained in relatively simple terms assuming rigid boundary conditions for the LC director at all substrates. Since the latter induce the LC director orientations within the *yz*-plane, we can describe the inhomogeneous LC alignment by the polar angle θ(**r**) as shown in Figure 4. For simplicity, we neglect a small possible deviation from the LC planar alignment on pristine rubbed PI and also use the so-called one-constant approximation of the orientational LC elasticity, which yields the Laplace equation Δθ(**r**) = 0 for the equilibrium distribution of the LC director [3].

In the homogeneous background area of the LC cell, hybrid LC alignment takes place as the LC is anchored in-plane by rubbed PI at one substrate, θ = π/2 at *z* = 0, and vertically by chromolane at the other substrate, θ = 0 at *z* = *d*. The corresponding solution of the Laplace equation reads

[3]
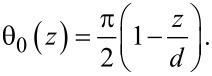


At the FIB-patterned areas, a periodic modulation of the LC polar angle is induced (see Figure 4). The corresponding solution of the Laplace equation can be found in terms of the Fourier expansion similar to that in [31]:

[4]
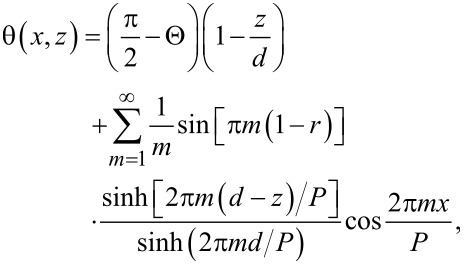


where the first term is independent of *x* and describes a smooth LC director rotation throughout the cell from θ = 0 at *z* = *d* to θ = π/2 − Θ at *z* = 0, and where

[2]
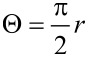


is the pretilt angle controlled by the duty factor *r*. The summation in the second term in Equation 4 is performed over a formally infinite number of oscillating harmonics that are localized near the patterned substrate and exponentially decay at a distance of a fraction of the pattern period *P*, i.e., on the submicrometer scale.

Consider now the two orthogonal linearly polarized light eigenstates propagating along the *z*-axis through the LC layer. Due to the LC birefringence, they acquire different optical paths:


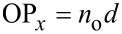


for the *x*-polarized state, and


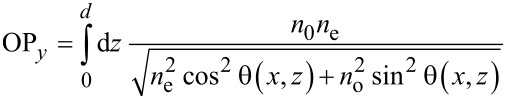


for the *y*-polarized state. For a relatively weak birefringence Δ*n* = *n*_e_ − *n*_o_, we can write the measured phase retardation approximately as

[5]
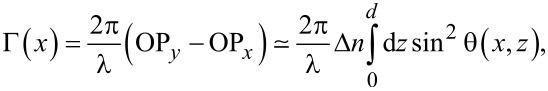


which is formally *x*-dependent. Substituting Equation 3 for the homogeneous background yields

[6]
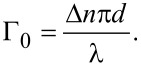


Above a stripe pattern, the first term in Equation 4, which is independent of *x*, provides the main contribution to the integral in Equation 5 while the remaining terms oscillating with *x* are negligible as they are localized in a submicrometer-thin surface layer. As we have recently shown [31–32], such layers being thicker when modulated with larger periodicity represent metasurfaces potentially hosting many interesting optical phenomena. In the present case, however, the smaller periods of 1–2 μm are deliberately chosen to minimise their optical contribution. Neglecting them and performing a simple integration we obtain Γ_p_ as independent of *x* and determine the analytical relation between the relative phase retardation and the pretilt angle:

[1]
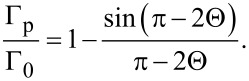


According to Equation 2, the latter is fully determined by the duty factor *r*. As seen from Figure 3a, the used model not only qualitatively explains the observations, but also yields dependences that fit remarkably well the experimental data accumulated for a large number of different samples.

Next, we determine the corresponding pretilt values by solving Equation 1 with respect to Θ for the measured values of the ratio Γ_p_/Γ_0_ and summarize them in Figure 3b. The simple dependence in Equation 2 fits the experimental data very well, which demonstrates that the above seemingly rough approximations yield quantitatively accurate results. Therefore, one can use Equation 2 as a precise guidance for predictable establishment of arbitrary local values of the pretilt angle.

The remarkable agreement between theory and experiment indicates, in particular, that above both the pristine and irradiated PI areas the LC anchoring is strong enough to be accounted within the rigid boundary conditions. One notes also that the dependence in Equation 1 used for extracting the pretilt values is strictly applicable only in terms of the one-constant approximation for the LC elasticity. However, it remains sufficiently accurate for realistically different elastic constants. Using values typical for the nematic E7 mixture with the ratio *K*_33_/*K*_11_ = 1.54 [33], we solve numerically the corresponding Euler–Lagrange equation for θ(*z*) with the boundary conditions θ(0) = π/2 − Θ and θ(*d*) = 0 on the LC cell substrates to evaluate Γ_p_, and with θ(0)= π/2 and θ(*d*) = 0 to evaluate Γ_0_. The corresponding relative retardation values appear to be very close to the analytical Equation 1 as one can see by comparing the solid and dashed curves in Figure 3a, where those dependences are represented as functions of *r* via Equation 2.

## Conclusion

In conclusion, we propose a digital method of precisely controlling the nematic LC pretilt angle at the aligning surface in the full range from 0° to 90°. The method is based on accurate mixing of two binary states (planar and vertical) of the LC director induced by pristine parts of the polymer layer and those treated with FIB. In this way, one can directly fabricate polymer substrates inducing arbitrary LC pretilt angle distributions and utilize them in versatile optical LC metasurfaces and electrically tunable metadevices, such as lenses, prisms and q-plates.
